# Maternal prenatal cholesterol levels predict offspring weight trajectories during childhood in the Norwegian Mother, Father and Child Cohort Study

**DOI:** 10.1186/s12916-023-02742-9

**Published:** 2023-02-06

**Authors:** Linn K. L. Øyri, Jacob J. Christensen, Sylvain Sebert, Magne Thoresen, Trond M. Michelsen, Stine M. Ulven, Hilde K. Brekke, Kjetil Retterstøl, Anne Lise Brantsæter, Per Magnus, Martin P. Bogsrud, Kirsten B. Holven

**Affiliations:** 1 Department of Nutrition, Institute of Basic Medical Sciences, University of Oslo, PO Box 1046, Blindern, 0317 Oslo, Norway; 2 Research Unit of Population Health, Faculty of Medicine, PO Box 5000, FI-90014 University of Oulu, Oulu, Finland; 3 Oslo Centre for Biostatistics and Epidemiology, Department of Biostatistics, Institute of Basic Medical Sciences, University of Oslo, PO Box 1122, Blindern, 0317 Oslo, Norway; 4 Department of Obstetrics, Oslo University Hospital Rikshospitalet, PO Box 4956, Nydalen, 0424 Oslo, Norway; 5 Institute of Clinical Medicine, Faculty of Medicine, University of Oslo, PO Box 1171, Blindern, 0318 Oslo, Norway; 6 The Lipid Clinic, Department of Endocrinology, Morbid Obesity and Preventive Medicine, Oslo University Hospital Aker, PO Box 4959, Nydalen, 0424 Oslo, Norway; 7 Division of Climate and Environmental Health, Department of Food Safety, Norwegian Institute of Public Health, PO Box 222, Skøyen, 0213 Oslo, Norway; 8 Centre for Fertility and Health, Norwegian Institute of Public Health, PO Box 222, Skøyen, 0213 Oslo, Norway; 9 Unit for Cardiac and Cardiovascular Genetics, Department of Medical Genetics, Oslo University Hospital Ullevål, PO Box 4956, Nydalen, 0424 Oslo, Norway; 10 Norwegian National Advisory Unit on Familial Hypercholesterolemia, Department of Endocrinology, Morbid Obesity and Preventive Medicine, Oslo University Hospital Aker, PO Box 4959, Nydalen, 0424 Oslo, Norway

**Keywords:** MoBa, MBRN, Cholesterol, Weight trajectories, Parental negative control study

## Abstract

**Background:**

Numerous intrauterine factors may affect the offspring’s growth during childhood. We aimed to explore if maternal and paternal prenatal lipid, apolipoprotein (apo)B and apoA1 levels are associated with offspring weight, length, and body mass index from 6 weeks to eight years of age. This has previously been studied to a limited extent.

**Methods:**

This parental negative control study is based on the Norwegian Mother, Father and Child Cohort Study and uses data from the Medical Birth Registry of Norway. We included 713 mothers and fathers with or without self-reported hypercholesterolemia and their offspring. Seven parental metabolites were measured by nuclear magnetic resonance spectroscopy, and offspring weight and length were measured at 12 time points. Data were analyzed by linear spline mixed models, and the results are presented as the interaction between parental metabolite levels and offspring spline (age).

**Results:**

Higher maternal total cholesterol (TC) level was associated with a larger increase in offspring body weight up to 8 years of age (0.03 ≤ *P*_interaction_ ≤ 0.04). Paternal TC level was not associated with change in offspring body weight (0.17 ≤ *P*_interaction_ ≤ 0.25). Higher maternal high-density lipoprotein cholesterol (HDL-C) and apoA1 levels were associated with a lower increase in offspring body weight up to 8 years of age (0.001 ≤ *P*_interaction_ ≤ 0.005). Higher paternal HDL-C and apoA1 levels were associated with a lower increase in offspring body weight up to 5 years of age but a larger increase in offspring body weight from 5 to 8 years of age (0.01 ≤ *P*_interaction_ ≤ 0.03). Parental metabolites were not associated with change in offspring height or body mass index up to 8 years of age (0.07 ≤ *P*_interaction_ ≤ 0.99).

**Conclusions:**

Maternal compared to paternal TC, HDL-C, and apoA1 levels were more strongly and consistently associated with offspring body weight during childhood, supporting a direct intrauterine effect.

**Supplementary Information:**

The online version contains supplementary material available at 10.1186/s12916-023-02742-9.

## Background

Obesity is associated with several non-communicable diseases such as cardiovascular disease, diabetes, and cancer. Children and adolescents with obesity have increased risk of being obese as adults. The worldwide prevalence of overweight and obesity in children and adolescents aged 5–19 years has increased from 4% in 1975 to 18% in 2016 [1]. It is now well established that prenatal exposure to numerous lifestyle and environmental factors associate with the offspring’s risk of disease in adulthood [2, 3]. Offspring body mass index (BMI) during childhood can be affected by parental factors through genetics, epigenetics, and intrauterine- and postnatal environment [4, 5]. In recent years, maternal pre-pregnancy BMI [5, 6], gestational weight gain [6], gestational dietary pattern [5, 7], and gestational diabetes [5] have been positively associated with offspring BMI during childhood.

Maternal cholesterol levels during pregnancy might also affect offspring growth during childhood, which has previously been studied to a limited extent [8–13]. This is relevant since about 30% of Norwegian and American women of reproductive age have elevated total cholesterol (TC) level [14, 15]. Additionally, the levels of low-density lipoprotein cholesterol (LDL-C) with its attached apolipoprotein (apo)B and high-density lipoprotein cholesterol (HDL-C) with its attached apoA1 increase substantially during pregnancy [16]. This facilitates vital transport of maternal cholesterol from LDL and HDL to the fetus across the placenta [17], as the fetal endogenous cholesterol production is low during the first half of pregnancy [18].

Cholesterol is needed in cell membranes and for the synthesis of sex hormones and glucocorticoids [19]. Cells depend on increased cholesterol synthesis and uptake to proliferate at a high pace [20], and cholesterol has been shown to stimulate cell growth signaling in vitro [21]. Moreover, a genome wide association study indicated that cholesterol biosynthesis is important for fetal growth [22]. Epidemiological studies in humans also suggest a link between cholesterol metabolism and parameters for growth. For example, obesity is positively associated with LDL-C level and negatively associated with HDL-C level [23]. Interestingly, studies have found inverse associations between age-standardized height and LDL-C level in children [24] and in adults [25] and between height and cardiovascular diseases [26].

Our hypothesis was that high maternal cholesterol level causes higher offspring cholesterol level and hence increased growth during childhood. Parental negative control studies can be used to explore causality [27–30]. A strong association between maternal cholesterol level and offspring growth and no or a much weaker association between paternal cholesterol level and offspring growth may indicate that there is a causal maternal intrauterine effect. An important assumption is that maternal and paternal cholesterol levels are affected equally by sources of bias or confounding. A strong association between the cholesterol level of both parents and offspring growth may indicate that the associations are caused by parental genotypes or shared familial confounding factors. We tested our hypothesis by exploring if maternal and paternal prenatal lipid, apoB, and apoA1 levels are associated with offspring weight, length, and BMI from 6 weeks to 8 years of age.

## Methods

### Subjects and study design

The Norwegian Mother, Father and Child Cohort Study (MoBa) is a population-based pregnancy cohort study conducted by the Norwegian Institute of Public Health [31]. Participants were recruited from all over Norway from 1999 to 2008. The women consented to participation in 41% of the invited pregnancies. The cohort now includes approximately 114,500 children, 95,200 mothers, and 75,200 fathers. Over 90% of the mothers were born in Norway. Blood samples were obtained from both parents during pregnancy [32], and the parents answered questionnaires during pregnancy and after birth.

We used data from nine questionnaires completed around gestational week 17 (questionnaires for mothers and fathers), gestational week 22, 6 months after birth, and 1.5, 3, 5, 7, and 8 years after birth (questionnaires for mothers). The questionnaires covered background information, lifestyle, illnesses, and other health-related factors. Data from the Medical Birth Registry of Norway (MBRN) were linked to the MoBa database using the unique personal identification number assigned to all residents in Norway [33]. The MBRN is a national health registry containing information about all births in Norway. The current study is based on version 11 of the quality-assured data files released for research in September 2018.

The sample in this exploratory sub-study has been described previously [34]. In brief, we excluded parents who had not completed the three questionnaires during pregnancy, who had a multiple pregnancy, miscarriage or stillbirth. We included 397 mothers with self-reported hypercholesterolemia or use of lipid-lowering treatment the last 6 months before pregnancy, and their partners and offspring, and 319 fathers with self-reported use of lipid-lowering treatment the last 6 months before pregnancy and their partners and offspring. We included these subjects to ensure a wide range in parental cholesterol levels. Thus, 713 mother-father-offspring trios were included, as both parents had hypercholesterolemia or used lipid-lowering treatment in three trios. None of the mothers and presumably all the fathers who used lipid-lowering treatment before pregnancy also used lipid-lowering treatment in the period around blood sampling.

### MoBa questionnaires and MBRN

Parental age (years), offspring age (years), sex (female/male), birth weight (kg), and length (cm) measured by health professionals, and gestational age (weeks) were obtained from MBRN. Offspring weight and length at ages 6 weeks and 3 and 6 months were obtained from the questionnaire completed 6 months after birth; measurements at ages 8 months and 1 and 1.5 years were obtained from the questionnaire completed 1.5 years after birth; measurements at ages 2 and 3 years were obtained from the questionnaire completed 3 years after birth; and the measurements at ages 5, 7, and 8 years were obtained from questionnaires at the respective ages. Weight and height are measured by health professionals at routine visits at public health centers from birth to 5 years of age and in the school health service up to 8 years of age in Norway [35]. The parents were asked to refer to these measurements from 6 weeks to 1.5 year, whereas this was not specified from 2 to 8 years of age. Maternal pre-pregnancy and paternal prenatal body weight (kg), parental height (cm), education (< 12, 12, 13–16, ≥ 17 years), and prenatal smoking (yes/no) were obtained from the questionnaire completed by mothers around gestational week 17. Maternal body weight (kg) at the end of pregnancy was obtained from the questionnaire completed 6 months after birth. BMI (kg/m^2^), gestational weight gain (kg), and Ponderal index (g/cm^3^) were calculated based on the above mentioned questionnaires. Maternal gestational weight gain was also categorized according to the IOM guidelines [36]. Offspring weight, length, and BMI for age corresponding to < − 3 or > 3 *z*-scores according to WHO growth references [37] were replaced with missing. BMI was calculated before the outliers were replaced with missing. Using this method, 1% of weight, 2% of length, and 1% of BMI measurements were replaced with missing. Parental self-reported use of lipid-lowering treatment (yes/no) from 6 months before the pregnancy to gestational week 17 and history of hypercholesterolemia were obtained from the questionnaires completed around gestational week 17.

### Blood samples

Non-fasting blood samples were obtained in mean (standard deviation [SD], min-max) gestational week 19 [1, 13–27] among the mothers and 19 [2, 13–41] among the fathers [34]. TC, LDL-C, HDL-C, triglycerides (TG), apoB, apoA1, apoB/apoA1 ratio, and glucose levels were measured in EDTA plasma samples by nuclear magnetic resonance spectroscopy at the accredited laboratory Nightingale Health in Finland [38].

### Statistics

The statistical analyses were performed in R (version 4.1.3) [39] with RStudio [40]. Descriptive data are presented as frequencies (%) for categorical variables and as mean (SD) or median (interquartile range) for continuous variables, unless otherwise noted. Weight, length, and BMI for age *z*-scores according to WHO growth references [37] were calculated using the ‘addWGSR’ package. Mothers who contributed with two pregnancies (*n* = 32) are represented with the first pregnancy in baseline characteristics and with both pregnancies in the mixed model analyses. We first fitted linear mixed models to explore the association between parental prenatal metabolites and offspring weight, length, and Ponderal index at birth using the ‘lme4’ package. In the models with maternal metabolites as exposure, we included maternal metabolite level (continuous), maternal pre-pregnancy BMI (continuous), maternal prenatal smoking (dichotomous), participation time (dichotomous), paternal metabolite level (continuous), and offspring sex (dichotomous) as fixed effects and id as random effect. Participation time was included to pick up the mothers who contributed with two pregnancies, who were given the same id to account for within-subject variance. In the models with paternal metabolites as exposure, we included paternal metabolite level (continuous), paternal prenatal BMI (continuous), paternal prenatal smoking (dichotomous), participation time (dichotomous), maternal metabolite level (continuous), and offspring sex (dichotomous) as fixed effects and id as random effect. It is not recommended to adjust for gestational age, which may lay on the causal pathway between parental metabolites and offspring birthweight [41].

To model growth over time, we then fitted linear mixed models with linear splines to explore the association between parental prenatal metabolites and offspring weight, length, and BMI from 6 weeks to 8 years of age (measured 11 times) using the ‘lme4’ package and the ‘splines::bs’ function. We placed knots at age 9 months (adiposity peak) and 5 years (adiposity rebound) for weight, length, and BMI for age [42]. The regression lines between the knots and a loess line are shown in Additional file 1: Figure S1. Directed acyclic graphs were drawn prior to the analyses to identify probable and clinically relevant confounders (Additional file 2: Figure S2). We performed three maternal and three paternal models. Maternal model 1 included maternal metabolite level (continuous), participation time (dichotomous), offspring spline (age [continuous]), and the interaction between maternal metabolite level and offspring spline (age) as fixed effects and id and offspring age (continuous) as random effects. Maternal model 2 included model 1 plus maternal pre-pregnancy BMI (continuous), maternal prenatal smoking (dichotomous), and offspring sex (dichotomous). Maternal model 3 included model 2 plus paternal metabolite level (continuous). Paternal model 1 included paternal metabolite level (continuous), participation time (dichotomous), offspring spline (age [continuous]), and the interaction between paternal metabolite level and offspring spline (age) as fixed effects and id and offspring age (continuous) as random effects. Paternal model 2 included model 1 plus paternal prenatal BMI (continuous), smoking (dichotomous) and offspring sex (dichotomous). Paternal model 3 included model 2 plus maternal metabolite level (continuous).

Because a significant (*P*-value < 0.05) interaction effect would indicate that the association between parental metabolite level and offspring growth (weight, length, or BMI) varies with the children’s age, we also stratified the data by the children’s age to present regression coefficients (*β* with 95% confidence intervals [CI]) for parental metabolites between the knots (≥ 6 weeks and < 9 months vs. ≥ 9 months and < 5 years vs. ≥ 5 years and ≤ 8 years). The distribution of the residuals was checked and found satisfactory.

We conducted sensitivity analyses with additional adjustment for variables with uncertain relevance; parental age, education, glucose level, maternal dietary intake of total fat (E%) and polyunsaturated/saturated fatty acids (ratio), paternal use of lipid-lowering treatment, and offspring gestational age. The method for lipid-lowering treatment adjustment has been described previously [34]. Briefly, the parental metabolites were divided by the effect of statin treatment found in another study [43], i.e., TC/0.773, LDL-C/0.651, HDL-C/0.992, TG/0.823, apoB/0.772, apoA1/0.974, and apoB/apoA1 ratio/0.794. Additionally, we performed stratified analyses in (1) mothers with self-reported hypercholesterolemia or lipid-lowering treatment use and (2) mothers without self-reported hypercholesterolemia or lipid-lowering treatment use.

## Results

### Baseline characteristics

Characteristics of the population have been published previously [34]. In brief, we included 713 mother-father-child trios, of which 32 mothers contributed with two pregnancies. The mothers’ mean age at delivery was 32 years, mean pre-pregnancy BMI was 25 kg/m^2^, and mean gestational weight gain was 14 kg (Table 1). Twenty percent of the mothers had lower gestational weight gain, and 48% had higher gestational weight gain than the IOM guidelines [36]. The fathers’ mean age at delivery was 35 years and mean BMI was 27 kg/m^2^. Among the mothers and fathers, 68% and 56% had higher education, 7% and 16% smoked during pregnancy, and 2% and 5% had diabetes mellitus, respectively. Half of the children were girls. The median gestational age was 40 weeks (Table 1), and the children were mean 3.6 (SD 0.5) kg and 50.5 (SD 1.9) cm at birth (data not shown). This corresponded to a weight for age z-score of mean 1.0 (SD 0.6) according to the WHO child growth standards (data not shown). The children’s anthropometric measures from birth to 8 years of age are shown in Table 2.

**Table 1:** Baseline characteristics of the 681 mother-father-newborn trios

	Mothers	***n***^a^	Fathers	***n***^a^	Newborns	***n***^a^
**Age (years), mean (SD)**	31.7 (4.5)	681	34.8 (6.1)	681	0	681
**BMI**^**b**^**(kg/m**^**2**^**), mean (SD)**	25.2 (5.0)	669	27.0 (3.9)	663		
**Gestational weight gain (kg), mean (SD)**	14.4 (6.3)	586				
**Education (years), *n *(%)**		661		653		
**< 12**	46 (7.0)		58 (8.9)			
**12**	168 (25.4)		231 (35.4)			
**13–16**	274 (41.5)		202 (30.9)			
≥ **17**	173 (26.2)		162 (24.8)			
**Smoking during pregnancy, *n *(%)**	50 (7.4)	680	108 (16.0)	676		
**Female, *n *(%)**					342 (50.2)	681
**Gestational age (weeks), median (IQR)**					40 (39–40)	681
**Ponderal index**^**c **^**(g/cm**^**3**^**), mean (SD)**					2.8 (0.3)	635
**Lipid-lowering treatment**^**d**^**, *n *(%)**	0	681	305 (44.8)	681		
**TC (mmol/l), mean (SD)**	5.5 (1.3)	679	3.8 (0.9)	680	1.4 (0.5)	676
**LDL-C (mmol/l), mean (SD)**	2.0 (0.7)	679	1.5 (0.5)	680	0.3 (0.2)	676
**HDL-C (mmol/l), mean (SD)**	1.8 (0.3)	679	1.1 (0.2)	680	0.8 (0.2)	676
**TG (mmol/l), median (IQR)**	1.4 (1.0–1.7)	679	1.1 (0.9–1.5)	680	0.3 (0.3–0.4)	676
**apoB (g/l), mean (SD)**	1.0 (0.3)	679	0.8 (0.2)	680	0.3 (0.1)	676
**apoA1 (g/l), mean (SD)**	1.8 (0.2)	679	1.3 (0.1)	680	0.9 (1.1)	676
**apoB/apoA1 (ratio), mean (SD)**	0.6 (0.1)	679	0.6 (0.1)	680	0.4 (0.1)	676
**Glucose (mmol/l), mean (SD)**	3.5 (0.7)	678	3.8 (1.4)	676	1.2 (1.5)	592

*SD* standard deviation, *BMI* body mass index, *IQR* interquartile range, *TC* total cholesterol, *LDL-C* low-density lipoprotein cholesterol, *HDL-C* high-density lipoprotein cholesterol, *TG* triglycerides, *apo* apolipoprotein

^a^Includes first pregnancy only (*n* = 32 mothers in our sample contributed with two pregnancies, yielding a total of *n* = 713 pregnancies); ^b^pre-pregnancy for mothers, prenatal for fathers (around gestational week 17); ^c^at birth; ^d^at the time of blood sampling

**Table 2:** Anthropometric measures in offspring aged 0–8 years

Age	Weight (kg)	***n***	Length (cm)	***n***	BMI (kg/m^**2**^)	***n***
Birth	3.6 (0.5)	701	50.4 (1.9)	668	14.2 (1.3)	678
6 weeks	5.0 (0.7)	624	56.6 (2.3)	479	15.5 (1.5)	496
3 months	6.3 (0.8)	624	61.8 (2.3)	609	16.6 (1.6)	616
6 months	7.9 (1.0)	632	67.6 (2.3)	612	17.3 (1.6)	628
8 months	8.8 (1.1)	524	70.8 (2.5)	492	17.5 (1.5)	520
1 year	10.0 (1.2)	524	76.2 (2.7)	507	17.1 (1.4)	523
1.5 year	11.0 (1.3)	287	80.7 (3.1)	282	16.9 (1.3)	288
2 years	12.9 (1.6)	275	88.3 (3.7)	272	16.5 (1.4)	264
3 years	15.2 (1.8)	385	96.6 (3.9)	378	16.2 (1.3)	364
5 years	20.0 (2.7)	324	113.0 (5.1)	329	15.7 (1.5)	323
7 years	25.3 (3.8)	398	126.0 (5.2)	408	16.0 (1.9)	399
8 years	28.6 (4.5)	313	132.0 (5.7)	323	16.5 (2.0)	315

Data are presented as mean (standard deviation)

*BMI* body mass index

### Parental prenatal metabolites and offspring anthropometric measures at birth

Parental TC, LDL-C, HDL-C, TG, apoB, apoA1, and apoB/apoA1 ratio levels were not significantly associated with offspring weight, length, or Ponderal index at birth (− 0.41 ≤ β ≤ 0.58, 0.19 ≤ *P* ≤ 0.96) (Additional file 3: Table S1).

### Parental prenatal metabolites and offspring growth trajectories up to 8 years of age

**Fig. 1:**
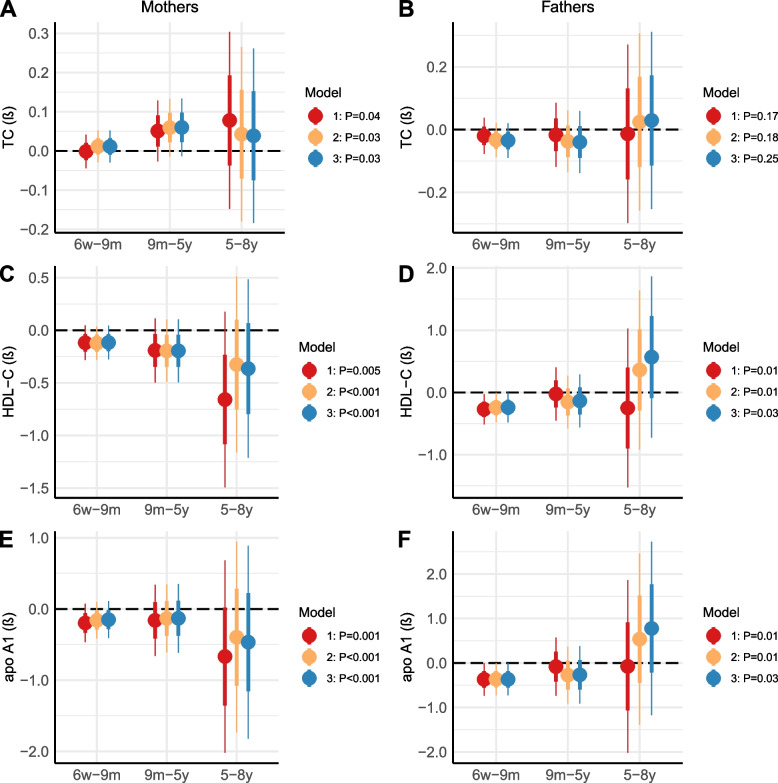
**A**–**F** Associations between parental prenatal metabolites and offspring weight up to 8 years of age. Results from linear spline mixed model analyses. Knots were placed at age 9 months and 5 years. *P*-values from the interaction between maternal or paternal metabolite level and offspring spline (age). The data were stratified to present regression coefficients (β) with one standard error on each side (thick error bars) and 95% confidence intervals (thin error bars) between the knots. Model 1 was adjusted for offspring age. Model 2 was adjusted for maternal or paternal BMI, smoking and offspring sex and age. Model 3 was adjusted for maternal or paternal metabolite level, BMI, smoking and offspring sex and age. TC, total cholesterol; HDL-C, high-density lipoprotein cholesterol; apo, apolipoprotein; w, weeks; m, months; y, years

Models 1–3 gave similar results. Higher maternal TC level was associated with a larger increase in offspring body weight up to 8 years of age (0.03 ≤ *P*_interaction_ ≤ 0.04; 6 weeks–9 months, 0.00 ≤ *β* ≤ 0.01; 9 months–5 years, 0.05 ≤ *β* ≤ 0.06; 5–8 years, 0.04 ≤ *β* ≤ 0.08; Fig. 1 and Additional file 4: Table S2, Additional file 5: Table S3 and Additional file 6: Table S4). Paternal TC level was not significantly associated with change in offspring body weight up to 8 years of age (0.17 ≤ *P*_interaction_ ≤ 0.25). Higher maternal HDL-C level was associated with a lower increase in offspring body weight up to 8 years of age (0.001 ≤ *P*_interaction_ ≤ 0.005; 6 weeks–9 months, *β* = –0.12 for all; 9 months–5 years, − 0.20 ≤ *β* ≤ − 0.19; 5–8 years, − 0.66 ≤ *β* ≤ − 0.33). Higher paternal HDL-C level was associated with a lower increase in offspring body weight up to 5 years of age, but a larger increase in offspring body weight from 5 to 8 years of age in model 2 and 3 (0.01 ≤ *P*_interaction_ ≤ 0.03; 6 weeks–9 months, − 0.27 ≤ *β* ≤ − 0.24; 9 months–5 years, − 0.15 ≤ β ≤ − 0.02; 5–8 years, − 0.25 ≤ *β* ≤ 0.57). Higher maternal apoA1 level was associated with a lower increase in offspring body weight up to 8 years of age (*P*_interaction_ ≤ 0.001 for all; 6 weeks–9 months, − 0.20 ≤ *β* ≤ − 0.15; 9 months–5 years, − 0.16 ≤ *β* ≤ − 0.13; 5–8 years, − 0.67 ≤ *β* ≤ − 0.40). Higher paternal apoA1 level was associated with a lower increase in offspring body weight up to 5 years of age, but a larger increase in offspring body weight from 5 to 8 years of age in model 2 and 3 (0.01 ≤ *P*_interaction_ ≤ 0.03; 6 weeks–9 months, *β* = − 0.37 for all; 9 months–5 years, − 0.27 ≤ *β* ≤ − 0.08; 5–8 years, − 0.08 ≤ *β* ≤ 0.78). Scatter plots of the associations between parental metabolites and offspring weight at specific ages are shown in Additional file 7: Figure S3.

Parental LDL-C, TG, apoB, and apoB/apoA1 ratio were not significantly associated with change in offspring body weight up to 8 years of age (0.10 ≤ *P*_interaction_ ≤ 0.85). None of the parental metabolites were significantly associated with change in offspring length or BMI up to 8 years of age (0.07 ≤ *P*_interaction_ ≤ 0.99; Additional file 4: Table S2, Additional file 5: Table S3 and Additional file 6: Table S4). The sensitivity analyses gave similar results as presented above (data not shown). Results from analyses stratified by maternal self-reported hypercholesterolemia are shown in Additional file 8: Table S5 and Additional file 9: Table S6. In mothers with hypercholesterolemia, higher maternal TC, TG, and apoB levels were associated with a larger increase in offspring body weight up to 8 years of age (0.01 ≤ *P*_interaction_ ≤ 0.04). In mothers without hypercholesterolemia, higher maternal HDL-C and apoA1 levels were associated with a lower increase in offspring body weight from 9 months to 8 years of age (*P*_interaction_ < 0.001 for both), higher maternal apoB/apoA1 ratio was associated with a larger increase in offspring body weight from 9 months to 8 years of age (*P*_interaction_ = 0.001), while maternal TG level was inconsistently associated with offspring body weight up to 8 years of age (*P*_interaction_ < 0.001).

## Discussion

To our knowledge, this is the first study showing that maternal prenatal TC level was positively associated with offspring body weight and maternal prenatal HDL-C and apoA1 levels were negatively associated with offspring body weight during the first 8 years of life. There was no evidence that paternal TC level was associated with offspring body weight. The negative associations between paternal HDL-C and apoA1 levels with offspring body weight were weaker and inconsistent compared to the associations between maternal HDL-C and apoA1 levels with offspring body weight. These results support the hypothesis that the associations may be caused by maternal intrauterine effects and not genetics or shared familial environmental factors.

The results from the three maternal models were highly consistent, suggesting that the effects were independent of maternal BMI and smoking and paternal metabolite levels. The regression coefficients for children between 9 months and 8 years of age indicated that offspring weight was 40–80 g higher for every 1 mmol/l higher maternal TC level and that offspring weight was 190–660 g lower for every 1 mmol/l higher maternal HDL-C level. However, the clinical or biological relevance of the current findings should be further explored. We included parents with or without self-reported hypercholesterolemia, and this may have affected the generalizability of the results. The stratified analyses indicate that there may be different associations between maternal TC and HDL-C with offspring body weight in mothers with hypercholesterolemia than in mothers without. However, the lower statistical power and the selection bias in the stratified analyses should be kept in mind. The mean maternal prenatal TC level in the current study (5.5 mmol/l) was similar to the mean maternal prenatal TC level in two other MoBa studies (5.4 mmol/l) which did not recruit subjects based on cholesterol levels [44, 45] and much lower than the mean prenatal TC level in women with familial hypercholesterolemia (9.1 mmol/l) [46].

Our hypothesis was that high maternal cholesterol level causes higher offspring cholesterol level and hence increased growth during childhood. We measured maternal cholesterol levels around gestational week 19. The fetus depends on maternal supply of cholesterol up to this time, after which fetal endogenous production of cholesterol has been shown to appear [18]. The role of maternal cholesterol when the fetus is able to synthesize its own cholesterol is unclear; however, a strong correlation between maternal and fetal TC level in a small sample of fetuses younger than 6 months has been shown [47]. We have previously found a positive association between maternal prenatal HDL-C and newborn HDL-C level [34]. Hence, maternal mid-pregnancy TC and HDL-C levels probably affect fetal TC and HDL-C levels but also possibly offspring TC and HDL-C later in childhood [48, 49]. High offspring LDL-C level may increase and high offspring HDL-C level may decrease the supply of cholesterol to peripheral cells [50], which in theory may affect the offspring’s development or epigenetic pattern, and subsequent body growth [5, 20–22]. This possible effect was mostly apparent from 9 months of age according to the data presented in the current study. It should be noted that the function of HDL may differ in fetuses and adults [51], and newborns have a higher proportion of HDL-C to LDL-C than their mothers [34].

The association between maternal prenatal cholesterol levels and offspring growth beyond 2 years of age [10] has scarcely been studied previously. In accordance with other studies [11–13], we did not find a significant association between maternal prenatal cholesterol levels and offspring BMI during childhood. This might be related to the relatively small regression coefficients for the association between maternal cholesterol levels and offspring weight. Furthermore, studies have found a positive association between maternal prenatal TC level with offspring adiposity at 4 years of age [8] and fat percentage at 5–6 years of age [9], and inconsistent associations between maternal prenatal TG level with offspring growth during childhood have been reported [9–13]. This inconsistency might partly be explained by differences in adjustment for maternal BMI and glucose level. None of the above mentioned studies included paternal lipids or repeated measurements of offspring growth.

We found no evidence that parental cholesterol levels were associated with offspring height. Height is greatly influenced by genetics among Europeans [52]. Some studies have found an inverse association between LDL-C and height [24, 25]. One of these studies showed that single-nucleotide polymorphisms affecting height were positively associated with LDL-C [25]. As maternal cholesterol levels were associated with offspring weight and not height, our findings might be mediated though effect of cholesterol on adiposity. Cholesterol is important for cell proliferation [19, 20] and fetal growth [22] and has interestingly been shown to activate the mechanistic target of rapamycin (mTOR) complex 1 [21]. This complex promotes cell proliferation [21] and adipogenesis [53]. mTOR is expressed in the placenta and activation has been linked to fetal growth [54]. Fetal adipose tissue has been shown to appear and develop during the second trimester, and the number of fat cells is relatively stable from early in life. Fetal life may therefore be critical for adipogenesis [55].

Furthermore, we suggest that the associations between maternal cholesterol levels and offspring growth might be mediated through various hormones. Cholesterol is required for the production of sex hormones and glucocorticoids [19] and has been positively associated with levels of cortisol [56], leptin [57], and insulin [58], which are highly metabolically active hormones. Initiation of lipid-lowering treatment in non-pregnant subjects has been shown to decrease levels of testosterone [59] and leptin [60] and to decrease insulin secretion [61]. Cortisol [62] and insulin [63] promote adipogenesis. Testosterone [64], estrogen [64], and leptin [65] have been shown to stimulate growth hormone, but it is unclear whether cholesterol level has a direct effect on growth hormone levels. In fact, elevated fetal leptin and insulin levels have been suggested to predispose to postnatal adiposity [66]. Hence, it is possible that high prenatal LDL-C level might increase and high prenatal HDL-C level might decrease offspring adipogenesis and growth during childhood through mTOR complex 1 or metabolically active hormones. These possible mechanisms should be further explored.

Another possible mechanism could be that an unhealthy maternal diet leading to maternal hypercholesterolemia can be transmitted to the offspring and cause increased growth. Maternal high fat diet around gestation has been associated with increased offspring body weight later in life in animals [67]. Maternal dietary pattern during pregnancy has also been associated with offspring BMI during childhood in humans [5, 7]. The current associations between maternal cholesterol levels and offspring weight did not change when adjusting for maternal dietary fat intake in sensitivity analyses. Still, maternal HDL-C level may be a marker of a healthy lifestyle [68], which might exert beneficial effects for the offspring in the pre- and postnatal phases, causing lower body weight during childhood.

Strengths in the current study include the use of a parental negative control design, the large number of blood samples from both parents during pregnancy, and detailed data on a number of confounding variables from both MoBa and MBRN. The wide ranges in parental cholesterol levels [34] make this sample well-suited to study the association between parental cholesterol levels and offspring anthropometric measures, which were measured 12 times. The study also has some limitations. Around half of the sample in the current study was lost to follow-up at age 8 years. Parents who were lost to follow-up have been shown to be younger, have higher BMI, lower education, and a higher smoking prevalence than parents who continued to respond [69]. Several variables were self-reported, including offspring weight and height after birth, parental weight, height, smoking prevalence, education, and use of lipid-lowering treatment. Another Norwegian cohort study has found substantial agreement between self-reported and measured BMI [70]. Self-reported smoking has been validated against plasma cotinine level in the MoBa study [71]. There may be residual confounding by environmental exposures such as maternal diet and paternal use of lipid-lowering treatment, even if we tried to adjust for this. Finally, the blood samples were non-fasting, however there are only minor postprandial changes in TC, LDL-C, HDL-C, apoB, and apoA1 [72].

## Conclusions

The novel findings in the present study include that maternal prenatal TC level was positively associated with offspring body weight, and maternal prenatal HDL-C and apoA1 levels were negatively associated with offspring body weight during childhood. Paternal prenatal TC level was not associated with offspring body weight, and paternal compared to maternal prenatal HDL-C and apoA1 levels were weaker and inconsistently associated with offspring body weight during childhood. These results indicate that the associations may be caused by maternal intrauterine effects. Future studies should aim to replicate the findings and explore the associations between maternal prenatal lipids and offspring body composition.

## Supplementary Information

**Additional file 1: Figure S1.** Growth charts with linear splines.

**Additional file 2: Figure S2.** Directed acyclic graphs for the association between parental metabolites and offspring growth up to 8 years of age.

**Additional file 3: Table S1.** Associations between parental prenatal metabolites and offspring anthropometric measures at birth.

**Additional file 4: Table S2.** Associations between parental prenatal metabolites and offspring anthropometric measures from 6 weeks to 8 years of age (model 1).

**Additional file 5: Table S3.** Associations between parental prenatal metabolites and offspring anthropometric measures from 6 weeks to 8 years of age (model 2).

**Additional file 6: Table S4.** Associations between parental prenatal metabolites and offspring anthropometric measures from 6 weeks to 8 years of age (model 3).

**Additional file 7: Figure S3.** Scatter plots of the associations between parental prenatal metabolites and offspring weight at specific ages.

**Additional file 8: Table S5.** Associations between maternal prenatal metabolites and offspring anthropometric measures from 6 weeks to 8 years of age in mothers with hypercholesterolemia (*n* = 378).

**Additional file 9: Table S6.** Associations between maternal prenatal metabolites and offspring anthropometric measures from 6 weeks to 8 years of age in mothers without hypercholesterolemia (*n* = 303).

## Data Availability

Data from the Norwegian Mother, Father and Child Cohort Study and the Medical Birth Registry of Norway used in this study are managed by the national health register holders in Norway (Norwegian Institute of Public Health) and can be made available to researchers, provided approval from the Regional Committees for Medical and Health Research Ethics (REC), compliance with the EU General Data Protection Regulation (GDPR) and approval from the data owners. The consent given by the participants does not open for storage of data on an individual level in repositories or journals. Researchers who want access to data sets for replication should apply through https://helsedata.no. Access to data sets requires approval from The Regional Committee for Medical and Health Research Ethics in Norway and an agreement with MoBa.

## Abbreviations

Apo: Apolipoprotein
BMI: Body mass index
E%: Percentage of energy
HDL-C: High-density lipoprotein cholesterol
LDL-C: Low-density lipoprotein cholesterol
MBRN: Medical Birth Registry of Norway
MoBa: The Norwegian Mother, Father and Child Cohort Study
mTOR: Mechanistic target of rapamycin
SD: Standard deviation
TC: Total cholesterol
TG: Triglycerides
