# Supplementary material for: Maternal prenatal cholesterol levels predict offspring weight trajectories during childhood in the Norwegian Mother, Father and Child Cohort Study
Source: BMC Med. 2023 Feb 6;21:43. doi: 10.1186/s12916-023-02742-9 (PMC9903496; doi:10.1186/s12916-023-02742-9)
Supplement: Supplementary file 1 — Additional file 1: Figure S1. Growth charts with linear splines. [file 12916_2023_2742_MOESM1_ESM.pdf]

Additional file 1: Figure S1. Growth charts with linear splines.

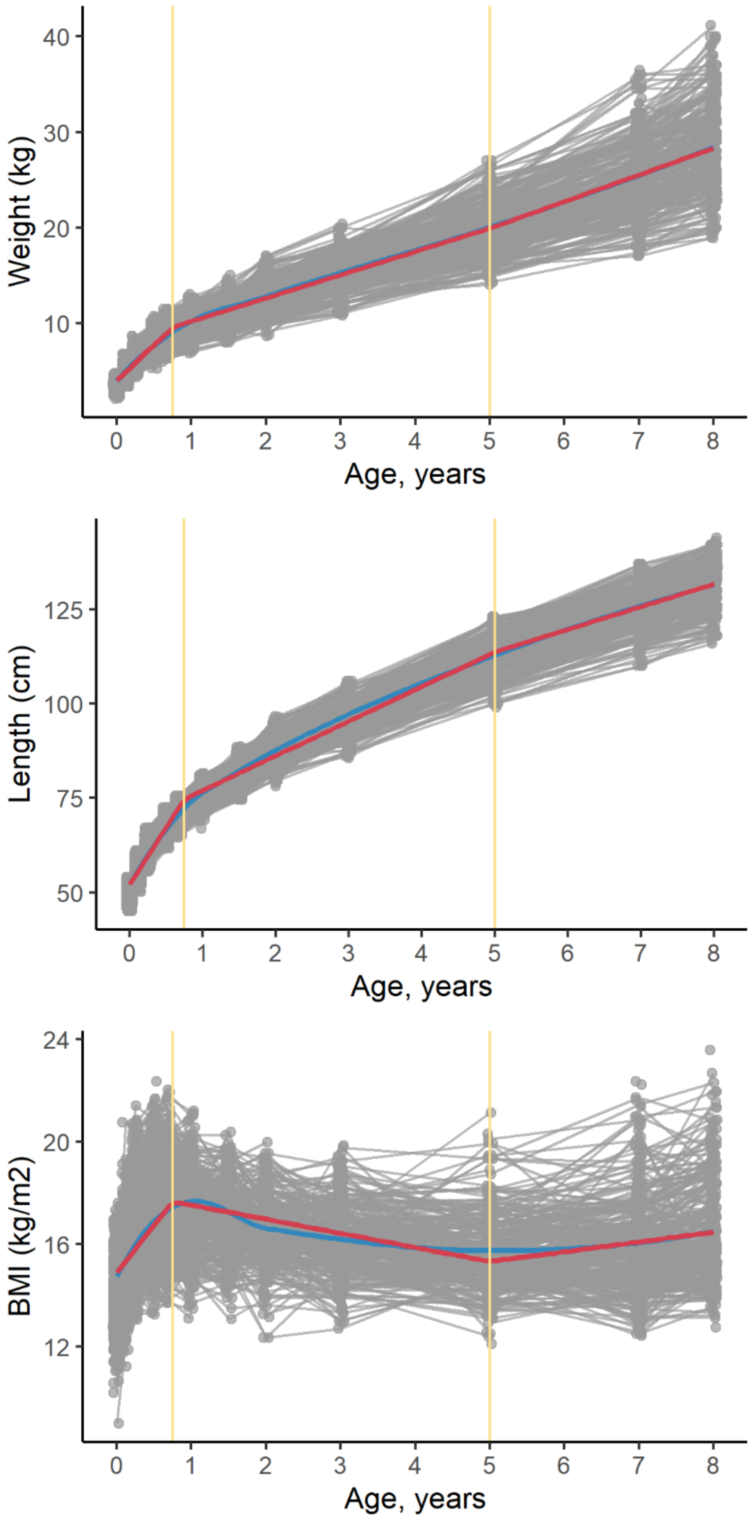

Red, linear splines with knots at age 9 months and 5 years; blue, loess lines; grey, individual growth curves.
