# Supplementary material for: Maternal prenatal cholesterol levels predict offspring weight trajectories during childhood in the Norwegian Mother, Father and Child Cohort Study
Source: BMC Med. 2023 Feb 6;21:43. doi: 10.1186/s12916-023-02742-9 (PMC9903496; doi:10.1186/s12916-023-02742-9)
Supplement: Supplementary file 2 — Additional file 2: Figure S2. Directed acyclic graphs for the association between parental metabolites and offspring growth up to 8 years of age. [file 12916_2023_2742_MOESM2_ESM.pdf]

Additional file 2: Figure S2. Directed acyclic graphs for the association between parental metabolites and offspring growth up to 8 years of age.

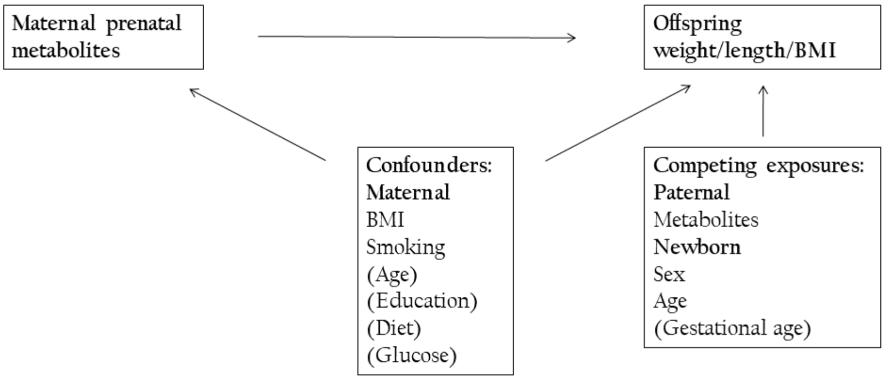

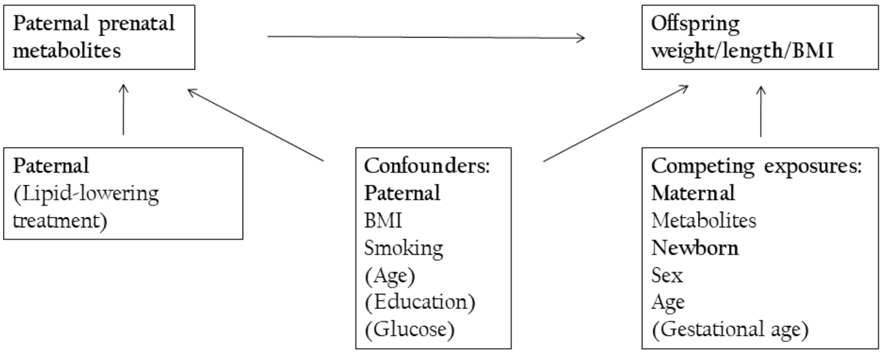

The variables in parentheses are used in sensitivity analyses only. Lipid-lowering treatment was not a potential confounder for mothers as none of the mothers used lipid-lowering treatment in the period around blood sampling.
