# Supplementary material for: Maternal prenatal cholesterol levels predict offspring weight trajectories during childhood in the Norwegian Mother, Father and Child Cohort Study
Source: BMC Med. 2023 Feb 6;21:43. doi: 10.1186/s12916-023-02742-9 (PMC9903496; doi:10.1186/s12916-023-02742-9)
Supplement: Supplementary file 3 — Additional file 3: Table S1. Associations between parental prenatal metabolites and offspring anthropometric measures at birth. [file 12916_2023_2742_MOESM3_ESM.pdf]

Additional file 3: Table S1. Associations between parental prenatal metabolites and offspring anthropometric measures at birth.

Exposure		Weight (kg)				Length (cm)				Ponderal index (g/cm^3)			
		**β**	**CI low**	**CI high**	**P-value**	**β**	**CI low**	**CI high**	**P-value**	**β**	**CI low**	**CI high**	**P-value**
Maternal	TC, mmol/l	-0.01	-0.04	0.02	0.47	-0.01	-0.12	0.10	0.88	-0.01	-0.02	0.01	0.42
Maternal	LDL-C, mmol/l	-0.02	-0.07	0.03	0.51	-0.01	-0.21	0.20	0.96	-0.01	-0.04	0.01	0.33
Maternal	HDL-C, mmol/l	-0.08	-0.19	0.04	0.20	-0.26	-0.72	0.20	0.26	0.00	-0.06	0.07	0.92
Maternal	TG, mmol/l	0.02	-0.05	0.09	0.49	0.12	-0.14	0.39	0.36	0.00	-0.04	0.03	0.91
Maternal	apoB, g/l	-0.01	-0.15	0.13	0.87	0.10	-0.43	0.64	0.71	-0.03	-0.10	0.04	0.45
Maternal	apoA1, g/l	-0.12	-0.31	0.06	0.20	-0.36	-1.10	0.37	0.33	0.00	-0.10	0.10	0.93
Maternal	apoB/apoA1, ratio	0.06	-0.20	0.31	0.66	0.45	-0.53	1.43	0.37	-0.05	-0.19	0.08	0.43
Paternal	TC, mmol/l	0.01	-0.03	0.05	0.47	0.04	-0.12	0.20	0.64	0.00	-0.02	0.02	0.80
Paternal	LDL-C, mmol/l	0.04	-0.03	0.11	0.26	0.14	-0.15	0.42	0.34	-0.01	-0.04	0.03	0.76
Paternal	HDL-C, mmol/l	-0.10	-0.28	0.08	0.26	-0.32	-1.03	0.39	0.38	-0.05	-0.15	0.04	0.29
Paternal	TG, mmol/l	0.00	-0.08	0.08	0.95	-0.12	-0.45	0.21	0.47	0.02	-0.02	0.07	0.30
Paternal	apoB, g/l	0.09	-0.10	0.29	0.35	0.18	-0.60	0.96	0.65	0.02	-0.08	0.12	0.71
Paternal	apoA1, g/l	-0.08	-0.35	0.18	0.54	-0.41	-1.47	0.64	0.44	-0.04	-0.19	0.10	0.54
Paternal	apoB/apoA1, ratio	0.19	-0.10	0.48	0.19	0.58	-0.55	1.71	0.32	0.04	-0.11	0.20	0.58

Results are presented as regression coefficients (β) with 95 % confidence intervals (CI) and P-values from linear mixed model analyses. The models were adjusted for maternal or paternal metabolite level, BMI, smoking and offspring sex. TC, total cholesterol; LDL-C, low-density lipoprotein cholesterol; HDL-C, high-density lipoprotein cholesterol; TG, triglycerides, apo, apolipoprotein.
