# Supplementary material for: Maternal prenatal cholesterol levels predict offspring weight trajectories during childhood in the Norwegian Mother, Father and Child Cohort Study
Source: BMC Med. 2023 Feb 6;21:43. doi: 10.1186/s12916-023-02742-9 (PMC9903496; doi:10.1186/s12916-023-02742-9)
Supplement: Supplementary file 4 — Additional file 4: Table S2. Associations between parental prenatal metabolites and offspring anthropometric measures from 6 weeks to 8 years of age (model 1). [file 12916_2023_2742_MOESM4_ESM.pdf]

Additional file 4: Table S2. Associations between parental prenatal metabolites and offspring anthropometric measures from 6 weeks to 8 years of age (model 1).

Exposure		6 weeks-9 months			9 months-5 years			5-8 years			$P_{interaction}$
**Offspring weight (kg)**											
		**Estimate**	**CI low**	**CI high**	**Estimate**	**CI low**	**CI high**	**Estimate**	**CI low**	**CI high**	
Maternal	TC, mmol/l	0.00	-0.04	0.04	0.05	-0.03	0.13	0.08	-0.15	0.30	**0.04**
Maternal	LDL-C, mmol/l	0.02	-0.06	0.09	0.12	-0.03	0.26	0.18	-0.23	0.59	0.10
Maternal	HDL-C, mmol/l	-0.12	-0.28	0.05	-0.19	-0.50	0.11	-0.66	-1.49	0.18	**0.005**
Maternal	TG, mmol/l	-0.04	-0.13	0.06	0.06	-0.11	0.22	0.45	-0.06	0.96	0.85
Maternal	apoB, g/l	0.02	-0.19	0.22	0.29	-0.08	0.65	0.78	-0.32	1.88	0.28
Maternal	apoA1, g/l	-0.20	-0.46	0.07	-0.16	-0.66	0.34	-0.67	-2.02	0.68	**0.001**
Maternal	apoB/apoA1, ratio	0.10	-0.26	0.46	0.63	-0.02	1.29	1.81	-0.14	3.77	0.57
Paternal	TC, mmol/l	-0.02	-0.08	0.04	-0.02	-0.12	0.09	-0.01	-0.30	0.27	0.17
Paternal	LDL-C, mmol/l	-0.01	-0.11	0.09	-0.02	-0.20	0.16	-0.07	-0.57	0.43	0.22
Paternal	HDL-C, mmol/l	-0.27	-0.51	-0.03	-0.02	-0.45	0.40	-0.25	-1.52	1.03	**0.01**
Paternal	TG, mmol/l	0.01	-0.09	0.11	-0.05	-0.23	0.13	0.32	-0.22	0.86	0.39
Paternal	apoB, g/l	0.01	-0.27	0.29	-0.08	-0.57	0.42	0.30	-1.06	1.66	0.53
Paternal	apoA1, g/l	-0.37	-0.74	0.00	-0.08	-0.74	0.57	-0.08	-2.02	1.86	**0.01**
Paternal	apoB/apoA1, ratio	0.17	-0.23	0.58	-0.12	-0.83	0.60	0.37	-1.60	2.33	0.54
**Offspring length (cm)**											
**Exposure**		**6 weeks-9 months**			**9 months-5 years**			**5-8 years**			$P_{interaction}$
		**Estimate**	**CI low**	**CI high**	**Estimate**	**CI low**	**CI high**	**Estimate**	**CI low**	**CI high**	
Maternal	TC, mmol/l	-0.01	-0.14	0.12	0.07	-0.12	0.25	0.28	-0.09	0.65	0.20
Maternal	LDL-C, mmol/l	0.02	-0.21	0.26	0.15	-0.18	0.49	0.51	-0.15	1.18	0.19
Maternal	HDL-C, mmol/l	-0.14	-0.64	0.36	-0.39	-1.12	0.34	-0.21	-1.60	1.17	0.91
Maternal	TG, mmol/l	-0.17	-0.45	0.12	0.18	-0.23	0.58	0.39	-0.42	1.21	0.54
Maternal	apoB, g/l	-0.07	-0.68	0.54	0.45	-0.42	1.31	1.43	-0.34	3.21	0.22
Maternal	apoA1, g/l	-0.28	-1.10	0.54	-0.33	-1.52	0.86	0.26	-1.98	2.50	0.80
Maternal	apoB/apoA1, ratio	-0.06	-1.15	1.03	0.96	-0.61	2.53	2.60	-0.58	5.78	0.33
Paternal	TC, mmol/l	0.10	-0.08	0.27	-0.01	-0.26	0.23	0.38	-0.07	0.84	0.18
Paternal	LDL-C, mmol/l	0.22	-0.09	0.53	-0.02	-0.46	0.41	0.59	-0.21	1.40	0.09
Paternal	HDL-C, mmol/l	-0.45	-1.19	0.30	0.34	-0.71	1.38	0.72	-1.36	2.81	0.58
Paternal	TG, mmol/l	0.14	-0.18	0.46	-0.11	-0.55	0.34	0.30	-0.57	1.18	0.32
Paternal	apoB, g/l	0.66	-0.18	1.50	-0.22	-1.40	0.97	1.74	-0.47	3.96	0.14
Paternal	apoA1, g/l	-0.27	-1.39	0.86	0.38	-1.22	1.98	1.91	-1.21	5.04	0.94
Paternal	apoB/apoA1, ratio	1.01	-0.21	2.24	-0.71	-2.41	1.00	1.59	-1.65	4.83	0.07

Exposure		Offspring BMI (kg/m²)									
		6 weeks-9 months			9 months-5 years			5-8 years			$P_{interaction}$
		Estimate	CI low	CI high	Estimate	CI low	CI high	Estimate	CI low	CI high	
Maternal	TC, mmol/l	0.02	-0.06	0.11	0.05	-0.04	0.13	-0.04	-0.16	0.07	0.29
Maternal	LDL-C, mmol/l	0.05	-0.10	0.20	0.11	-0.04	0.27	-0.05	-0.26	0.17	0.24
Maternal	HDL-C, mmol/l	-0.13	-0.45	0.18	-0.10	-0.42	0.23	-0.67	-1.11	-0.22	0.35
Maternal	TG, mmol/l	0.06	-0.12	0.24	0.00	-0.18	0.18	0.32	0.05	0.59	0.60
Maternal	apoB, g/l	0.19	-0.20	0.58	0.24	-0.16	0.63	0.18	-0.40	0.76	0.70
Maternal	apoA1, g/l	-0.14	-0.66	0.37	-0.07	-0.60	0.46	-0.91	-1.64	-0.19	0.23
Maternal	apoB/apoA1, ratio	0.45	-0.24	1.15	0.52	-0.20	1.24	0.89	-0.14	1.92	0.76
Paternal	TC, mmol/l	-0.05	-0.16	0.06	0.05	-0.06	0.15	-0.11	-0.26	0.04	0.71
Paternal	LDL-C, mmol/l	-0.04	-0.23	0.16	0.08	-0.11	0.27	-0.24	-0.50	0.03	0.75
Paternal	HDL-C, mmol/l	-0.22	-0.67	0.24	0.20	-0.25	0.64	-0.16	-0.84	0.52	0.89
Paternal	TG, mmol/l	-0.07	-0.27	0.12	-0.03	-0.23	0.16	0.20	-0.09	0.49	0.10
Paternal	apoB, g/l	-0.15	-0.68	0.39	0.15	-0.37	0.67	-0.24	-0.97	0.48	0.58
Paternal	apoA1, g/l	-0.38	-1.07	0.31	0.35	-0.34	1.04	-0.28	-1.32	0.75	0.76
Paternal	apoB/apoA1, ratio	-0.05	-0.83	0.72	0.00	-0.75	0.75	-0.25	-1.30	0.80	0.75

Results from linear spline mixed model analyses. Knots were placed at age 9 months and 5 years. P-values from the interaction between maternal or paternal metabolite level and offspring spline(age). The data were stratified to present regression coefficients (β) with 95 % confidence intervals (CI) for parental metabolites between the knots. The models were adjusted for offspring age. TC, total cholesterol; LDL-C, low-density lipoprotein cholesterol; HDL-C, high-density lipoprotein cholesterol; TG, triglycerides, apo, apolipoprotein.
