# Supplementary material for: Maternal prenatal cholesterol levels predict offspring weight trajectories during childhood in the Norwegian Mother, Father and Child Cohort Study
Source: BMC Med. 2023 Feb 6;21:43. doi: 10.1186/s12916-023-02742-9 (PMC9903496; doi:10.1186/s12916-023-02742-9)
Supplement: Supplementary file 5 — Additional file 5: Table S3. Associations between parental prenatal metabolites and offspring anthropometric measures from 6 weeks to 8 years of age (model 2). [file 12916_2023_2742_MOESM5_ESM.pdf]

**Additional file 5: Table S3. Associations between parental prenatal metabolites and offspring anthropometric measures from 6 weeks to 8 years of age (model 2).**

| Exposure | | 6 weeks-9 months | | | 9 months-5 years | | | 5-8 years | | | $P_{interaction}$ |
|---|---|---|---|---|---|---|---|---|---|---|---|
| **Offspring weight (kg)** | | | | | | | | | | | |
| | | Estimate | CI low | CI high | Estimate | CI low | CI high | Estimate | CI low | CI high | |
| Maternal | TC, mmol/l | 0.01 | -0.03 | 0.05 | 0.06 | -0.01 | 0.13 | 0.04 | -0.18 | 0.26 | **0.03** |
| Maternal | LDL-C, mmol/l | 0.03 | -0.04 | 0.11 | 0.13 | 0.00 | 0.26 | 0.11 | -0.29 | 0.51 | 0.11 |
| Maternal | HDL-C, mmol/l | -0.12 | -0.28 | 0.04 | -0.20 | -0.49 | 0.10 | -0.33 | -1.16 | 0.51 | **<0.001** |
| Maternal | TG, mmol/l | 0.00 | -0.09 | 0.10 | 0.09 | -0.08 | 0.26 | 0.12 | -0.41 | 0.66 | 0.71 |
| Maternal | apoB, g/l | 0.09 | -0.10 | 0.28 | 0.34 | -0.01 | 0.69 | 0.33 | -0.76 | 1.43 | 0.31 |
| Maternal | apoA1, g/l | -0.16 | -0.42 | 0.10 | -0.13 | -0.61 | 0.35 | -0.40 | -1.74 | 0.94 | **<0.001** |
| Maternal | apoB/apoA1, ratio | 0.22 | -0.13 | 0.57 | 0.73 | 0.09 | 1.36 | 0.76 | -1.21 | 2.73 | 0.44 |
| Paternal | TC, mmol/l | -0.03 | -0.09 | 0.02 | -0.04 | -0.14 | 0.06 | 0.02 | -0.26 | 0.31 | 0.18 |
| Paternal | LDL-C, mmol/l | -0.03 | -0.13 | 0.07 | -0.05 | -0.22 | 0.13 | 0.03 | -0.47 | 0.52 | 0.23 |
| Paternal | HDL-C, mmol/l | -0.24 | -0.48 | -0.01 | -0.15 | -0.58 | 0.27 | 0.36 | -0.92 | 1.64 | **0.01** |
| Paternal | TG, mmol/l | -0.01 | -0.11 | 0.09 | -0.04 | -0.22 | 0.14 | -0.05 | -0.61 | 0.52 | 0.30 |
| Paternal | apoB, g/l | -0.08 | -0.35 | 0.19 | -0.13 | -0.61 | 0.36 | 0.08 | -1.28 | 1.45 | 0.57 |
| Paternal | apoA1, g/l | -0.37 | -0.72 | -0.01 | -0.27 | -0.91 | 0.36 | 0.54 | -1.39 | 2.46 | **0.01** |
| Paternal | apoB/apoA1, ratio | 0.06 | -0.33 | 0.46 | -0.07 | -0.77 | 0.63 | -0.23 | -2.21 | 1.75 | 0.61 |
| **Offspring length (cm)** | | | | | | | | | | | |
| **Exposure** | | **6 weeks-9 months** | | | **9 months-5 years** | | | **5-8 years** | | | $P_{interaction}$ |
| | | Estimate | CI low | CI high | Estimate | CI low | CI high | Estimate | CI low | CI high | |
| Maternal | TC, mmol/l | 0.03 | -0.10 | 0.15 | 0.07 | -0.10 | 0.25 | 0.24 | -0.13 | 0.60 | 0.19 |
| Maternal | LDL-C, mmol/l | 0.08 | -0.14 | 0.30 | 0.16 | -0.16 | 0.49 | 0.42 | -0.25 | 1.08 | 0.20 |
| Maternal | HDL-C, mmol/l | -0.22 | -0.70 | 0.26 | -0.42 | -1.15 | 0.30 | 0.20 | -1.20 | 1.60 | 0.90 |
| Maternal | TG, mmol/l | -0.01 | -0.30 | 0.27 | 0.27 | -0.15 | 0.69 | 0.04 | -0.83 | 0.91 | 0.65 |
| Maternal | apoB, g/l | 0.16 | -0.41 | 0.74 | 0.53 | -0.32 | 1.38 | 0.94 | -0.85 | 2.73 | 0.24 |
| Maternal | apoA1, g/l | -0.27 | -1.05 | 0.51 | -0.32 | -1.49 | 0.84 | 0.57 | -1.67 | 2.81 | 0.76 |
| Maternal | apoB/apoA1, ratio | 0.38 | -0.66 | 1.42 | 1.13 | -0.43 | 2.69 | 1.41 | -1.83 | 4.66 | 0.36 |
| Paternal | TC, mmol/l | 0.06 | -0.11 | 0.23 | -0.05 | -0.29 | 0.20 | 0.38 | -0.08 | 0.84 | 0.20 |
| Paternal | LDL-C, mmol/l | 0.15 | -0.15 | 0.44 | -0.07 | -0.50 | 0.36 | 0.61 | -0.21 | 1.42 | 0.09 |
| Paternal | HDL-C, mmol/l | -0.56 | -1.29 | 0.17 | 0.10 | -0.95 | 1.15 | 0.84 | -1.28 | 2.97 | 0.57 |
| Paternal | TG, mmol/l | 0.19 | -0.13 | 0.51 | -0.07 | -0.53 | 0.39 | 0.23 | -0.69 | 1.16 | 0.30 |
| Paternal | apoB, g/l | 0.55 | -0.27 | 1.36 | -0.27 | -1.46 | 0.91 | 1.68 | -0.57 | 3.93 | 0.17 |
| Paternal | apoA1, g/l | -0.43 | -1.52 | 0.65 | 0.02 | -1.57 | 1.60 | 1.93 | -1.22 | 5.07 | 0.91 |
| Paternal | apoB/apoA1, ratio | 1.00 | -0.19 | 2.19 | -0.60 | -2.32 | 1.11 | 1.47 | -1.85 | 4.80 | 0.09 |

| Exposure | | Offspring BMI ($kg/m^2$) | | | | | | | | | $P_{interaction}$ |
|---|---|---|---|---|---|---|---|---|---|---|---|
| | | 6 weeks-9 months | | | 9 months-5 years | | | 5-8 years | | | |
| | | Estimate | CI low | CI high | Estimate | CI low | CI high | Estimate | CI low | CI high | |
| Maternal | TC, mmol/l | 0.04 | -0.04 | 0.12 | 0.05 | -0.04 | 0.13 | -0.06 | -0.18 | 0.05 | 0.22 |
| Maternal | LDL-C, mmol/l | 0.07 | -0.08 | 0.21 | 0.11 | -0.04 | 0.26 | -0.08 | -0.29 | 0.14 | 0.24 |
| Maternal | HDL-C, mmol/l | -0.02 | -0.34 | 0.29 | -0.04 | -0.37 | 0.29 | -0.49 | -0.94 | -0.05 | 0.25 |
| Maternal | TG, mmol/l | 0.05 | -0.14 | 0.23 | -0.03 | -0.22 | 0.17 | 0.13 | -0.15 | 0.41 | 0.56 |
| Maternal | apoB, g/l | 0.04 | -0.48 | 0.55 | 0.21 | -0.19 | 0.61 | -0.07 | -0.64 | 0.51 | 0.67 |
| Maternal | apoA1, g/l | 0.37 | -0.32 | 1.07 | -0.01 | -0.53 | 0.52 | -0.77 | -1.48 | -0.06 | 0.14 |
| Maternal | apoB/apoA1, ratio | 0.20 | -0.18 | 0.59 | 0.44 | -0.29 | 1.17 | 0.32 | -0.72 | 1.35 | 0.86 |
| Paternal | TC, mmol/l | -0.07 | -0.18 | 0.04 | 0.04 | -0.07 | 0.15 | -0.08 | -0.23 | 0.07 | 0.78 |
| Paternal | LDL-C, mmol/l | -0.07 | -0.27 | 0.12 | 0.08 | -0.12 | 0.27 | -0.18 | -0.44 | 0.09 | 0.87 |
| Paternal | HDL-C, mmol/l | -0.14 | -0.60 | 0.33 | 0.26 | -0.20 | 0.72 | 0.28 | -0.40 | 0.96 | 0.89 |
| Paternal | TG, mmol/l | -0.16 | -0.36 | 0.05 | -0.09 | -0.29 | 0.11 | -0.08 | -0.38 | 0.23 | 0.12 |
| Paternal | apoB, g/l | -0.34 | -0.88 | 0.20 | 0.07 | -0.46 | 0.59 | -0.43 | -1.15 | 0.29 | 0.61 |
| Paternal | apoA1, g/l | -0.36 | -1.06 | 0.34 | 0.38 | -0.31 | 1.08 | 0.16 | -0.86 | 1.17 | 0.81 |
| Paternal | apoB/apoA1, ratio | -0.34 | -1.12 | 0.45 | -0.14 | -0.91 | 0.63 | -0.72 | -1.77 | 0.32 | 0.75 |

Results from linear spline mixed model analyses. Knots were placed at age 9 months and 5 years. P-values from the interaction between maternal or paternal metabolite level and offspring spline(age). The data were stratified to present regression coefficients (β) with 95 % confidence intervals (CI) for parental metabolites between the knots. The models were adjusted for maternal or paternal BMI, smoking and offspring sex and age. TC, total cholesterol; LDL-C, low-density lipoprotein cholesterol; HDL-C, high-density lipoprotein cholesterol; TG, triglycerides, apo, apolipoprotein.
