# Supplementary material for: Maternal prenatal cholesterol levels predict offspring weight trajectories during childhood in the Norwegian Mother, Father and Child Cohort Study
Source: BMC Med. 2023 Feb 6;21:43. doi: 10.1186/s12916-023-02742-9 (PMC9903496; doi:10.1186/s12916-023-02742-9)
Supplement: Supplementary file 6 — Additional file 6: Table S4. Associations between parental prenatal metabolites and offspring anthropometric measures from 6 weeks to 8 years of age (model 3). [file 12916_2023_2742_MOESM6_ESM.pdf]

Additional file 6: Table S4. Associations between parental prenatal metabolites and offspring anthropometric measures from 6 weeks to 8 years of age (model 3).

Offspring weight (kg)											
**Exposure**		**6 weeks-9 months**			**9 months-5 years**			**5-8 years**			**$P_{interaction}$**
		**Estimate**	**CI low**	**CI high**	**Estimate**	**CI low**	**CI high**	**Estimate**	**CI low**	**CI high**	
Maternal	TC, mmol/l	0.01	-0.03	0.05	0.06	-0.01	0.13	0.04	-0.18	0.26	**0.03**
Maternal	LDL-C, mmol/l	0.03	-0.04	0.11	0.13	0.00	0.26	0.10	-0.30	0.51	0.11
Maternal	HDL-C, mmol/l	-0.12	-0.28	0.04	-0.20	-0.49	0.10	-0.36	-1.21	0.48	**<0.001**
Maternal	TG, mmol/l	0.01	-0.09	0.10	0.10	-0.07	0.27	0.12	-0.41	0.66	0.70
Maternal	apoB, g/l	0.09	-0.10	0.28	0.34	0.00	0.69	0.32	-0.77	1.42	0.31
Maternal	apoA1, g/l	-0.15	-0.41	0.11	-0.13	-0.61	0.35	-0.47	-1.82	0.89	**<0.001**
Maternal	apoB/apoA1, ratio	0.22	-0.13	0.56	0.74	0.10	1.37	0.76	-1.21	2.74	0.44
Paternal	TC, mmol/l	-0.03	-0.09	0.02	-0.04	-0.14	0.06	0.03	-0.25	0.31	0.25
Paternal	LDL-C, mmol/l	-0.03	-0.13	0.06	-0.05	-0.23	0.12	0.03	-0.47	0.52	0.25
Paternal	HDL-C, mmol/l	-0.24	-0.48	0.00	-0.14	-0.56	0.29	0.57	-0.73	1.86	**0.03**
Paternal	TG, mmol/l	-0.01	-0.12	0.09	-0.05	-0.23	0.14	-0.09	-0.65	0.48	0.28
Paternal	apoB, g/l	-0.08	-0.35	0.19	-0.14	-0.63	0.34	0.04	-1.32	1.40	0.56
Paternal	apoA1, g/l	-0.37	-0.73	-0.01	-0.27	-0.91	0.38	0.78	-1.17	2.72	**0.03**
Paternal	apoB/apoA1, ratio	0.05	-0.34	0.45	-0.11	-0.81	0.59	-0.42	-2.40	1.56	0.57

Offspring length (cm)											
**Exposure**		**6 weeks-9 months**			**9 months-5 years**			**5-8 years**			**$P_{interaction}$**
		**Estimate**	**CI low**	**CI high**	**Estimate**	**CI low**	**CI high**	**Estimate**	**CI low**	**CI high**	
Maternal	TC, mmol/l	0.02	-0.10	0.14	0.08	-0.10	0.26	0.22	-0.15	0.58	0.18
Maternal	LDL-C, mmol/l	0.07	-0.14	0.29	0.16	-0.16	0.49	0.39	-0.27	1.05	0.19
Maternal	HDL-C, mmol/l	-0.21	-0.69	0.28	-0.43	-1.16	0.31	0.15	-1.27	1.57	0.89
Maternal	TG, mmol/l	-0.01	-0.29	0.27	0.27	-0.15	0.69	0.03	-0.84	0.90	0.66
Maternal	apoB, g/l	0.16	-0.41	0.73	0.53	-0.32	1.38	0.85	-0.94	2.64	0.24
Maternal	apoA1, g/l	-0.27	-1.05	0.52	-0.31	-1.49	0.86	0.46	-1.81	2.72	0.71
Maternal	apoB/apoA1, ratio	0.36	-0.68	1.40	1.16	-0.41	2.72	1.30	-1.95	4.54	0.37
Paternal	TC, mmol/l	0.05	-0.12	0.22	-0.07	-0.31	0.18	0.37	-0.09	0.83	0.22
Paternal	LDL-C, mmol/l	0.13	-0.16	0.43	-0.10	-0.53	0.34	0.59	-0.23	1.40	0.09
Paternal	HDL-C, mmol/l	-0.63	-1.36	0.10	0.05	-1.01	1.11	0.98	-1.17	3.13	0.65
Paternal	TG, mmol/l	0.18	-0.14	0.50	-0.08	-0.54	0.37	0.19	-0.74	1.11	0.29
Paternal	apoB, g/l	0.51	-0.30	1.32	-0.33	-1.52	0.85	1.55	-0.70	3.81	0.16
Paternal	apoA1, g/l	-0.55	-1.64	0.55	-0.12	-1.73	1.48	2.07	-1.11	5.25	0.99
Paternal	apoB/apoA1, ratio	1.00	-0.19	2.20	-0.65	-2.36	1.07	1.21	-2.12	4.54	0.08

Exposure		Offspring BMI (kg/m^2)									
		6 weeks-9 months			9 months-5 years			5-8 years			$P_{interaction}$
		**Estimate**	**CI low**	**CI high**	**Estimate**	**CI low**	**CI high**	**Estimate**	**CI low**	**CI high**	
Maternal	TC, mmol/l	0.04	-0.04	0.12	0.05	-0.04	0.13	-0.06	-0.18	0.05	0.21
Maternal	LDL-C, mmol/l	0.07	-0.08	0.22	0.11	-0.04	0.26	-0.07	-0.28	0.14	0.23
Maternal	HDL-C, mmol/l	-0.01	-0.33	0.30	-0.05	-0.38	0.28	-0.53	-0.98	-0.08	0.20
Maternal	TG, mmol/l	0.05	-0.14	0.23	-0.02	-0.22	0.17	0.13	-0.15	0.41	0.56
Maternal	apoB, g/l	0.21	-0.18	0.59	0.21	-0.19	0.61	-0.06	-0.64	0.51	0.67
Maternal	apoA1, g/l	0.06	-0.46	0.58	-0.02	-0.55	0.51	-0.82	-1.54	-0.10	0.11
Maternal	apoB/apoA1, ratio	0.38	-0.32	1.07	0.44	-0.29	1.17	0.35	-0.68	1.39	0.86
Paternal	TC, mmol/l	-0.07	-0.18	0.04	0.04	-0.07	0.15	-0.08	-0.23	0.07	0.74
Paternal	LDL-C, mmol/l	-0.07	-0.27	0.12	0.07	-0.12	0.27	-0.17	-0.44	0.09	0.86
Paternal	HDL-C, mmol/l	-0.11	-0.58	0.37	0.28	-0.18	0.74	0.39	-0.29	1.08	0.89
Paternal	TG, mmol/l	-0.16	-0.36	0.04	-0.09	-0.29	0.11	-0.10	-0.40	0.20	0.12
Paternal	apoB, g/l	-0.35	-0.89	0.19	0.06	-0.47	0.59	-0.45	-1.17	0.28	0.58
Paternal	apoA1, g/l	-0.33	-1.04	0.37	0.41	-0.29	1.11	0.29	-0.74	1.32	0.76
Paternal	apoB/apoA1, ratio	-0.36	-1.15	0.43	-0.17	-0.94	0.60	-0.81	-1.86	0.24	0.75

Results from linear spline mixed model analyses. Knots were placed at age 9 months and 5 years. P-values from the interaction between maternal or paternal metabolite level and offspring spline(age). The data were stratified to present regression coefficients (β) with 95 % confidence intervals (CI) for parental metabolites between the knots. The models were adjusted for maternal or paternal metabolite level, BMI, smoking and offspring sex and age. TC, total cholesterol; LDL-C, low-density lipoprotein cholesterol; HDL-C, high-density lipoprotein cholesterol; TG, triglycerides, apo, apolipoprotein.
