# Supplementary material for: Maternal prenatal cholesterol levels predict offspring weight trajectories during childhood in the Norwegian Mother, Father and Child Cohort Study
Source: BMC Med. 2023 Feb 6;21:43. doi: 10.1186/s12916-023-02742-9 (PMC9903496; doi:10.1186/s12916-023-02742-9)
Supplement: Supplementary file 7 — Additional file 7: Figure S3. Scatter plots of the associations between parental prenatal metabolites and offspring weight at specific ages. [file 12916_2023_2742_MOESM7_ESM.pdf]

Additional file 7: Figure S3. Scatter plots of the associations between parental prenatal metabolites and offspring weight at specific ages.

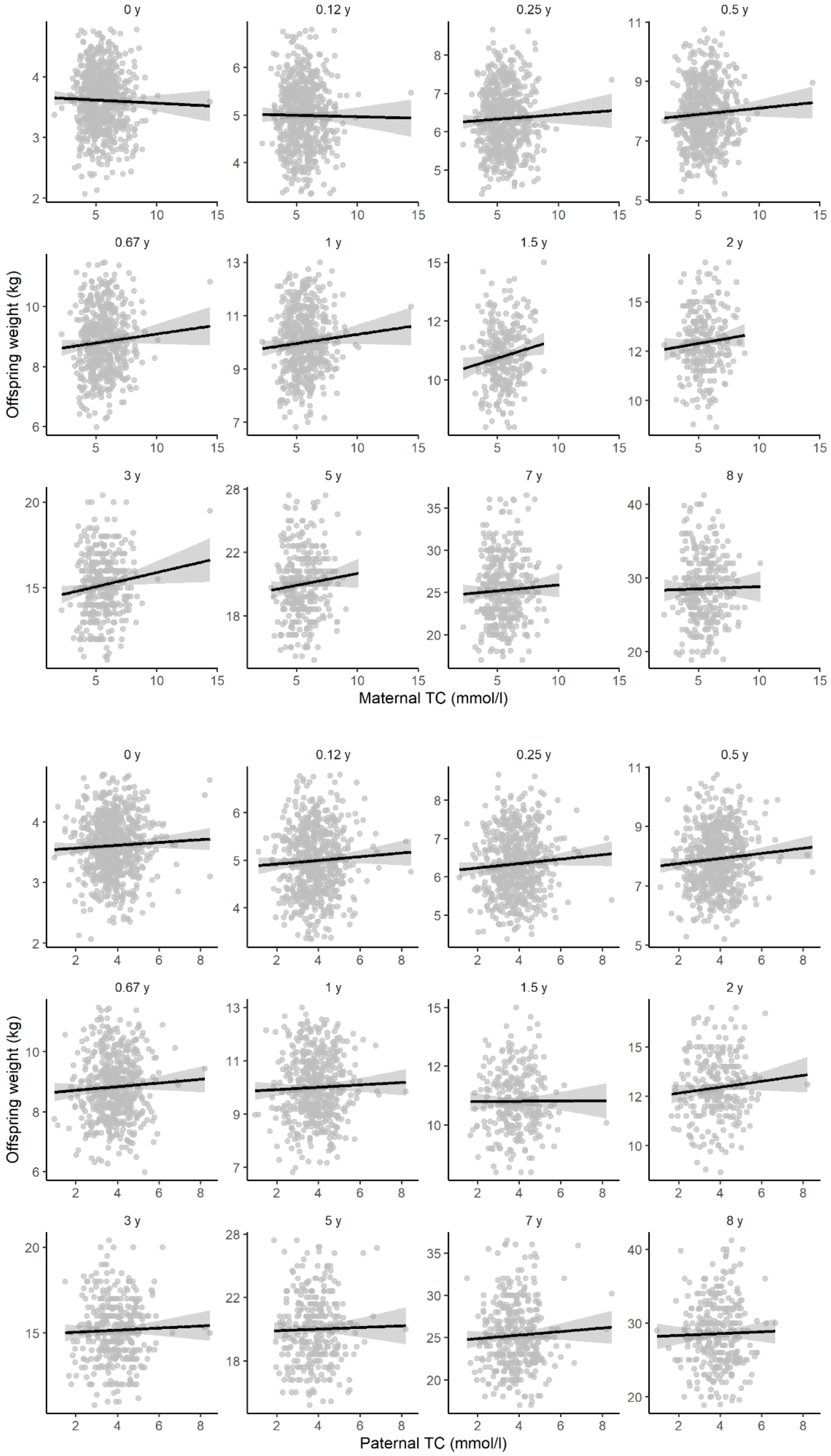

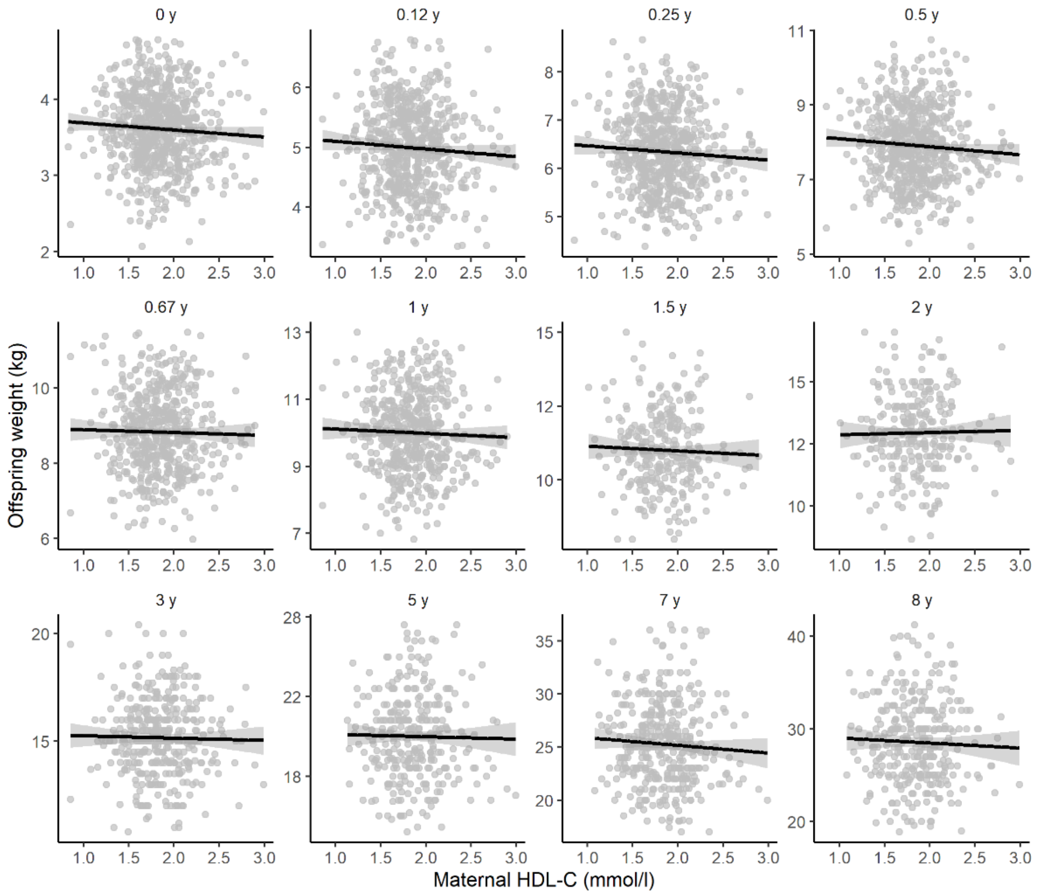
0 y
0.12 y
0.25 y
0.5 y
0.67 y
1 y
1.5 y
2 y
3 y
5 y
7 y
8 y
Offspring weight (kg)
Maternal HDL-C (mmol/l)

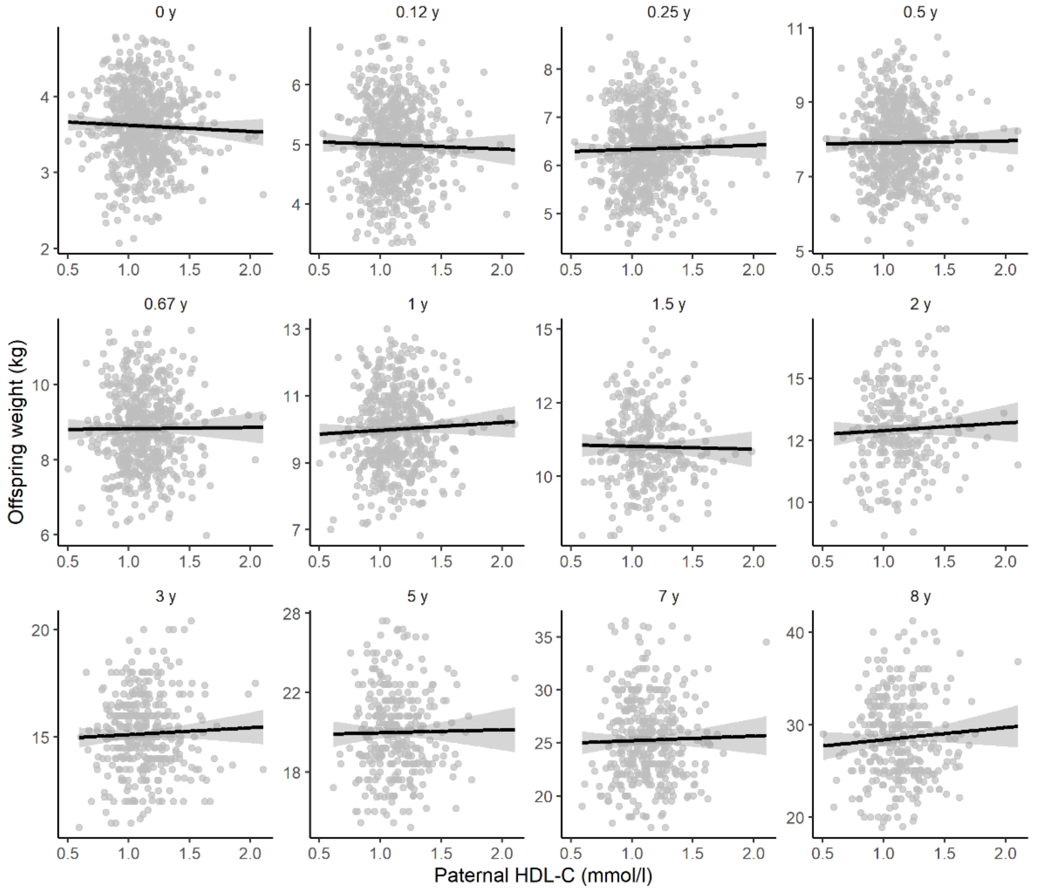
0 y
0.12 y
0.25 y
0.5 y
0.67 y
1 y
1.5 y
2 y
3 y
5 y
7 y
8 y
Offspring weight (kg)
Paternal HDL-C (mmol/l)

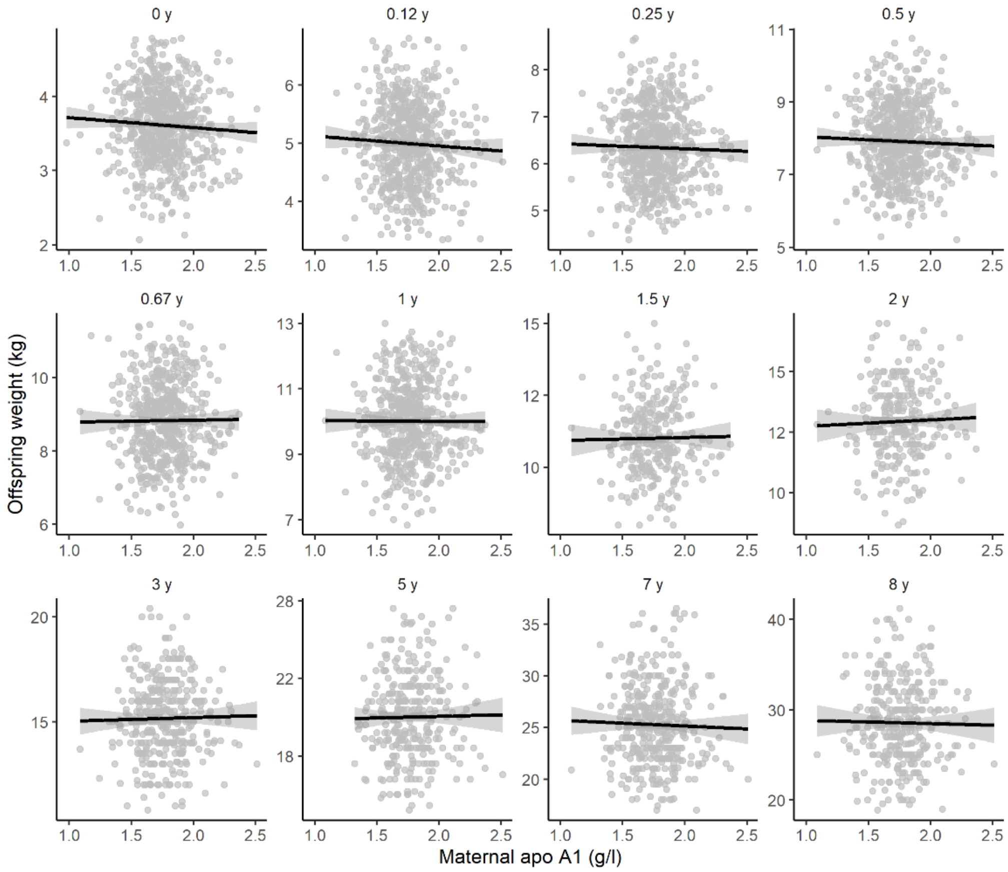

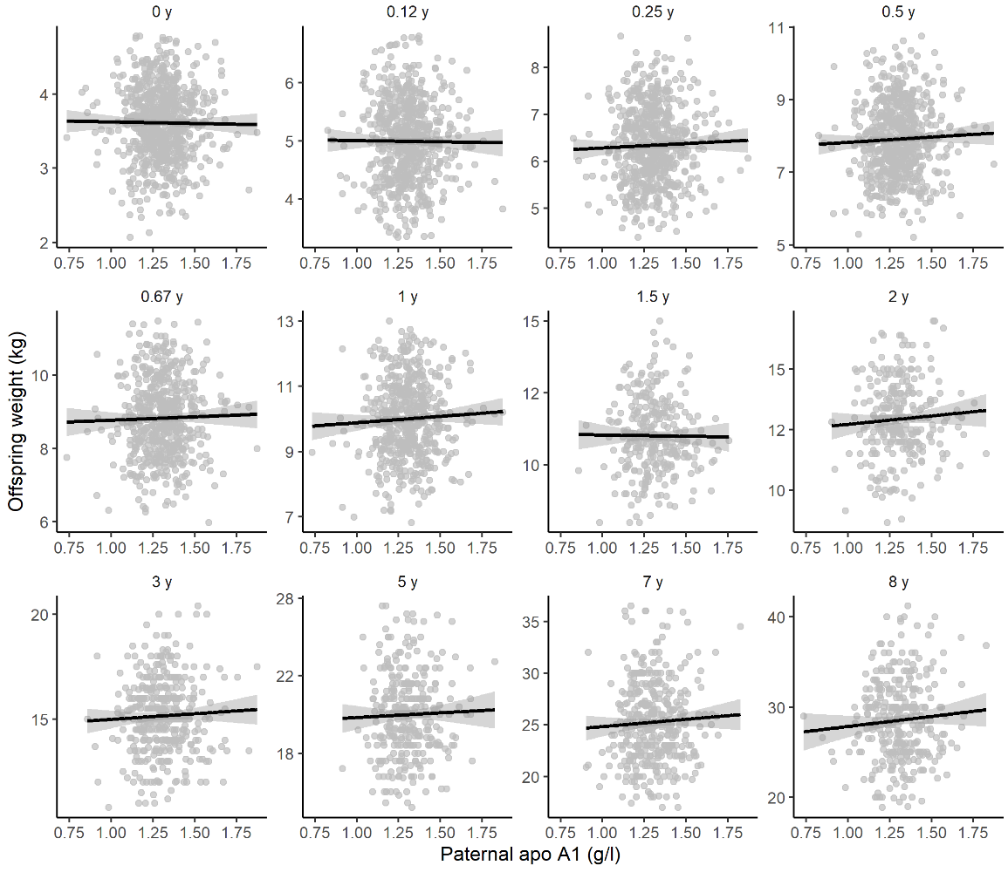

TC, total cholesterol; HDL-C, high-density lipoprotein cholesterol; apo, apolipoprotein.
