# Supplementary material for: Maternal prenatal cholesterol levels predict offspring weight trajectories during childhood in the Norwegian Mother, Father and Child Cohort Study
Source: BMC Med. 2023 Feb 6;21:43. doi: 10.1186/s12916-023-02742-9 (PMC9903496; doi:10.1186/s12916-023-02742-9)
Supplement: Supplementary file 8 — Additional file 8: Table S5. Associations between maternal prenatal metabolites and offspring anthropometric measures from 6 weeks to 8 years of age in mothers with hypercholesterolemia (n = 378). [file 12916_2023_2742_MOESM8_ESM.pdf]

Additional file 8: Table S5. Associations between maternal prenatal metabolites and offspring anthropometric measures from 6 weeks to 8 years of age in mothers with hypercholesterolemia (n = 378)[a].

Offspring weight (kg)										
Exposure	6 weeks-9 months			9 months-5 years			5-8 years			$P_{interaction}$
	Estimate	CI low	CI high	Estimate	CI low	CI high	Estimate	CI low	CI high	
TC, mmol/l	0.00	-0.05	0.06	0.05	-0.05	0.15	0.11	-0.19	0.41	**0.04**
LDL-C, mmol/l	0.03	-0.07	0.13	0.10	-0.07	0.28	0.21	-0.31	0.74	0.09
HDL-C, mmol/l	-0.23	-0.44	-0.03	-0.17	-0.56	0.21	-0.37	-1.40	0.66	0.75
TG, mmol/l	0.06	-0.07	0.18	0.06	-0.16	0.28	0.34	-0.33	1.02	**0.01**
apoB, g/l	0.12	-0.14	0.37	0.27	-0.20	0.73	0.77	-0.70	2.24	**0.01**
apoA1, g/l	-0.35	-0.70	0.01	-0.16	-0.82	0.50	-0.25	-1.98	1.47	0.48
apoB/apoA1, ratio	0.38	-0.06	0.82	0.58	-0.24	1.40	1.31	-1.20	3.81	0.08
**Offspring length (cm)**										
Exposure	6 weeks-9 months			9 months-5 years			5-8 years			$P_{interaction}$
	Estimate	CI low	CI high	Estimate	CI low	CI high	Estimate	CI low	CI high	
TC, mmol/l	-0.02	-0.19	0.14	0.09	-0.16	0.33	0.51	-0.01	1.02	**0.04**
LDL-C, mmol/l	-0.01	-0.30	0.28	0.18	-0.25	0.61	0.77	-0.12	1.66	0.10
HDL-C, mmol/l	-0.39	-1.02	0.24	-0.45	-1.37	0.48	0.77	-1.04	2.59	0.84
TG, mmol/l	0.09	-0.28	0.46	0.30	-0.24	0.83	0.32	-0.81	1.45	0.32
apoB, g/l	0.06	-0.71	0.83	0.57	-0.55	1.70	1.92	-0.57	4.40	0.06
apoA1, g/l	-0.70	-1.78	0.39	-0.41	-1.99	1.16	2.03	-1.00	5.06	0.37
apoB/apoA1, ratio	0.45	-0.90	1.79	1.24	-0.73	3.21	2.12	-2.16	6.41	0.24
**Offspring BMI (kg/m^2)**										
Exposure	6 weeks-9 months			9 months-5 years			5-8 years			$P_{interaction}$
	Estimate	CI low	CI high	Estimate	CI low	CI high	Estimate	CI low	CI high	
TC, mmol/l	0.01	-0.10	0.12	0.05	-0.06	0.16	-0.06	-0.21	0.10	0.40
LDL-C, mmol/l	0.05	-0.15	0.24	0.11	-0.08	0.30	-0.05	-0.33	0.22	0.41
HDL-C, mmol/l	-0.21	-0.62	0.21	-0.04	-0.45	0.36	-0.55	-1.09	-0.02	0.60
TG, mmol/l	0.05	-0.20	0.30	-0.05	-0.29	0.19	0.20	-0.15	0.56	0.60
apoB, g/l	0.16	-0.35	0.67	0.19	-0.32	0.70	0.06	-0.70	0.83	0.84
apoA1, g/l	-0.29	-1.01	0.42	-0.03	-0.71	0.66	-0.89	-1.80	0.01	0.46
apoB/apoA1, ratio	0.46	-0.43	1.35	0.39	-0.51	1.29	0.58	-0.72	1.89	0.94

Results from linear spline mixed model analyses. Knots were placed at age 9 months and 5 years. P-values from the interaction between maternal metabolite level and offspring spline(age). The data were stratified to present regression coefficients (β) with 95 % confidence intervals (CI) for parental metabolites between the knots. The models were adjusted for paternal metabolite level, maternal BMI, smoking and offspring sex and age. [a]Self-reported hypercholesterolemia or use of lipid-lowering treatment the last six months before pregnancy; TC, total cholesterol; LDL-C, low-density lipoprotein cholesterol; HDL-C, high-density lipoprotein cholesterol; TG, triglycerides, apo, apolipoprotein.
