# Supplementary material for: Maternal prenatal cholesterol levels predict offspring weight trajectories during childhood in the Norwegian Mother, Father and Child Cohort Study
Source: BMC Med. 2023 Feb 6;21:43. doi: 10.1186/s12916-023-02742-9 (PMC9903496; doi:10.1186/s12916-023-02742-9)
Supplement: Supplementary file 9 — Additional file 9: Table S6. Associations between maternal prenatal metabolites and offspring anthropometric measures from 6 weeks to 8 years of age in mothers without hypercholesterolemia (n = 303). [file 12916_2023_2742_MOESM9_ESM.pdf]

Additional file 9: Table S6. Associations between maternal prenatal metabolites and offspring anthropometric measures from 6 weeks to 8 years of age in mothers without hypercholesterolemia (n = 303)[a].

Offspring weight (kg)										
Exposure	6 weeks-9 months			9 months-5 years			5-8 years			$P_{interaction}$
	Estimate	CI low	CI high	Estimate	CI low	CI high	Estimate	CI low	CI high	
TC, mmol/l	0.02	-0.06	0.11	0.02	-0.13	0.17	-0.01	-0.48	0.47	0.14
LDL-C, mmol/l	0.06	-0.11	0.23	0.09	-0.21	0.39	0.04	-0.89	0.96	0.72
HDL-C, mmol/l	0.07	-0.19	0.32	-0.27	-0.76	0.22	-0.20	-1.71	1.30	**<0.001**
TG, mmol/l	-0.12	-0.28	0.05	0.09	-0.22	0.39	-0.10	-1.12	0.93	**<0.001**
apoB, g/l	0.01	-0.43	0.44	0.30	-0.44	1.05	0.05	-2.35	2.45	0.19
apoA1, g/l	0.08	-0.33	0.49	-0.35	-1.13	0.42	-0.51	-2.86	1.85	**<0.001**
apoB/apoA1, ratio	-0.21	-0.92	0.51	0.79	-0.51	2.09	0.37	-3.85	4.59	**0.001**
**Offspring length (cm)**										
Exposure	6 weeks-9 months			9 months-5 years			5-8 years			$P_{interaction}$
	Estimate	CI low	CI high	Estimate	CI low	CI high	Estimate	CI low	CI high	
TC, mmol/l	0.07	-0.18	0.32	-0.02	-0.39	0.36	0.07	-0.68	0.82	0.96
LDL-C, mmol/l	0.20	-0.28	0.68	-0.04	-0.76	0.69	0.22	-1.24	1.68	1.00
HDL-C, mmol/l	0.03	-0.74	0.79	-0.29	-1.51	0.94	-0.43	-2.76	1.91	0.40
TG, mmol/l	-0.29	-0.78	0.20	0.07	-0.70	0.83	-0.42	-1.99	1.16	0.37
apoB, g/l	0.13	-1.11	1.37	0.06	-1.78	1.90	0.15	-3.56	3.86	0.95
apoA1, g/l	0.06	-1.16	1.29	-0.28	-2.22	1.66	-0.73	-4.40	2.93	0.42
apoB/apoA1, ratio	-0.30	-2.42	1.81	0.09	-3.17	3.35	0.58	-5.89	7.04	0.76
**Offspring BMI (kg/m^2)**										
Exposure	6 weeks-9 months			9 months-5 years			5-8 years			$P_{interaction}$
	Estimate	CI low	CI high	Estimate	CI low	CI high	Estimate	CI low	CI high	
TC, mmol/l	0.09	-0.07	0.26	0.07	-0.11	0.25	-0.08	-0.33	0.17	0.90
LDL-C, mmol/l	0.12	-0.21	0.46	0.18	-0.17	0.54	-0.06	-0.54	0.42	1.00
HDL-C, mmol/l	0.30	-0.19	0.79	-0.08	-0.64	0.49	-0.47	-1.26	0.32	0.21
TG, mmol/l	0.01	-0.30	0.32	0.03	-0.31	0.37	0.18	-0.36	0.71	0.16
apoB, g/l	0.32	-0.54	1.18	0.40	-0.49	1.30	0.05	-1.20	1.31	0.73
apoA1, g/l	0.48	-0.31	1.27	-0.06	-0.97	0.86	-0.72	-1.96	0.52	0.32
apoB/apoA1, ratio	0.07	-1.32	1.47	0.76	-0.77	2.28	0.85	-1.35	3.04	0.38

Results from linear spline mixed model analyses. Knots were placed at age 9 months and 5 years. P-values from the interaction between maternal metabolite level and offspring spline(age). The data were stratified to present regression coefficients (β) with 95 % confidence intervals (CI) for parental metabolites between the knots. The models were adjusted for paternal metabolite level, maternal BMI, smoking and offspring sex and age. [a]No self-reported hypercholesterolemia or use of lipid-lowering treatment the last six months before pregnancy; TC, total cholesterol; LDL-C, low-density lipoprotein cholesterol; HDL-C, high-density lipoprotein cholesterol; TG, triglycerides, apo, apolipoprotein.
